# Taste receptor polymorphisms and longevity: a systematic review and meta-analysis

**DOI:** 10.1007/s40520-020-01745-3

**Published:** 2020-11-10

**Authors:** Danilo Di Bona, Alberto Malovini, Giulia Accardi, Anna Aiello, Giuseppina Candore, Anna Ferrario, Mattia E. Ligotti, Anna Maciag, Annibale A. Puca, Calogero Caruso

**Affiliations:** 1 Department of Emergency and Organ Transplantation, University of Bari-Aldo Moro, Bari, Italy; 2 Laboratory of Informatics and Systems Engineering for Clinical Research, Clinical Scientific Institutes Maugeri, 27100 Pavia, Italy; 3 Laboratory of Immunopathology and Immunosenescence, Department of Biomedicine, Neurosciences and Advanced Technologies, University of Palermo, Corso Tuköry, 211, 90134 Palermo, Italy; 4 Cardiovascular Research Unit, IRCCS MultiMedica, 20138 Milan, Italy; 5 Department of Medicine, Surgery and Dentistry “Scuola Medica Salernitana”, University of Salerno, Baronissi, SA Italy

**Keywords:** Immune-inflammatory responses, Longevity, Meta-analysis, Taste receptors

## Abstract

Bitter taste receptors (TAS2R) are involved in a variety of non-tasting physiological processes, including immune-inflammatory ones. Therefore, their genetic variations might influence various traits. In particular, in different populations of South Italy (Calabria, Cilento, and Sardinia), polymorphisms of TAS2R16 and TAS238 have been analysed in association with longevity with inconsistent results. A meta-analytic approach to quantitatively synthesize the possible effect of the previous variants and, possibly, to reconcile the inconsistencies has been used in the present paper. TAS2R38 variants in the Cilento population were also analysed for their possible association with longevity and the obtained data have been included in the relative meta-analysis. In population from Cilento no association was found between TAS2R38 and longevity, and no association was observed as well, performing the meta-analysis with data of the other studies. Concerning TAS2R16 gene, instead, the genotype associated with longevity in the Calabria population maintained its significance in the meta-analysis with data from Cilento population, that, alone, were not significant in the previously published study. In conclusion, our results suggest that TAS2R16 genotype variant is associated with longevity in South Italy.

## Introduction

The sensory structures for taste are the taste buds, groups of cells contained in goblet-shaped structures, papillae. The taste receptor cells occur in taste buds in groups of 50–150. Taste receptors are found on the upper surface of the tongue, soft palate, upper oesophagus, the cheek, and epiglottis. On average, there are 2000–8000 taste buds on the human tongue, therefore, hundreds of thousands of receptor cells. However, there is a great variety in their number. Healthy humans may have from three to several thousand taste buds per square centimetre on the tip of the tongue, and this variability contributes to differences in the taste sensations experienced by different people. Taste buds functionally perceive sweet, bitter, umami, sour, and salty. Salty and sour taste sensations are both detected through ion channels. Sweet, bitter, and umami tastes, instead, are detected by way of G protein-coupled taste receptors, i.e., taste 1 receptor members (TAS1Rs) for sweet tastants and umami tastants and taste 2 receptor members (TAS2Rs) for bitter tastants [1–3]. This apparently limited repertoire seems evolutionarily sufficient for the recognition of essential dietary elements and to avoid potential dietary threats with negative impact on physiology [4].

From the data of the literature, it emerges that the bitter taste receptors could behave like pleiotropic genes, whose products are used by various cells and have a signalling function on various unrelated targets. In fact, TAS2Rs have been detected in a large number of cells and tissues, and activation of these receptors produces a diverse range of biological responses under normal conditions. Bitter detection is probably just one of the functions performed by this group of receptors, which could have a more general role in the homeostasis of organisms. Many TAS2R transcripts have been detected in polymorphonuclear neutrophils and accumulating evidence suggests that TAS2R-mediated signalling contributes much to the innate immunity in the epithelia of the organs that are connected to the external environment. TAS2Rs also play secretion roles in the lower gastrointestinal tract, since can induce anion secretion in large intestine, as protective response against noxious irritants. Several studies have demonstrated the roles of bitter compounds in regulating vascular smooth muscle contractility. Several studies have begun to reveal that TAS2R can cause or contribute to disease in extraoral tissues. For example, the TAS238 genotype is related to the susceptibility, severity and prognosis of chronic rhinosinusitis, while the non-functional TAS2R38 show an increased risk of colorectal cancer in a population of European ancestry. Therefore, their genetic variations can profoundly influence various traits, in a way that it is only just beginning to understand [1].

Studies on Long Living Individuals (LLIs), i.e*.*, people > 90 years, allowed to identify specific genes and genotypes involved in pathways thought to influence human lifespan, pathways related to diet, immune-inflammatory responses including stress responses and DNA repair [5, 6]. Therefore, genetic variation in bitter taste receptor could directly affect successful ageing by modulating food preference during life [7]. Another possibility, not mutually exclusive, concerns the datum that taste receptors are claimed to be an integral component of antimicrobial immune-inflammatory responses, at least, in upper respiratory tract infections [8]. Therefore, it is not surprising that some studies have analysed the possible association of taste receptors polymorphisms with longevity [9–11].

Recently, Melis et al. [9] have studied a functional variant of the taste gene TAS2R38. They have compared results from 94 Sardinian LLIs with young and middle-aged Sardinians. In the LLIs cohort they found an increased frequency of subjects carrying the homozygous genotype for the functional variant PAV/PAV (PAV codes for Proline, Alanine, and Valine) and a decreased frequency of those having homozygous genotype for the non-functional form AVI/AVI (AVI codes for Alanine, Valine, and Isoleucine), as compared to those determined in the controls [9].

However, in a previous study on taste receptors polymorphisms and longevity, no association was detected with this variant in a population from Calabria (South Italy) [10]. In fact, using a tagging approach, the authors investigated the possible association of the common genetic variation at the three bitter taste gene clusters on chromosomes 5, 7, and 12 with longevity in a population ranging from 60 to 106 years of age. The final selection included 41 single nucleotide polymorphisms (SNPs) belonging to 20 genes. They found that some polymorphisms of TAS2R16, TAS2R4 and TAS2R5 were associated with longevity. After correction for multiple testing, only one SNP, rs978739 of TAS2R16, showed a statistically significant association with longevity [10].

On the other hand, Malovini et al. [11] did not confirm this association by comparing results from 410 LLIs to those from 553 young controls, in a population from another area of South Italy (Cilento). Statistical power calculations showed that the analysed cohort was sufficiently powered to replicate the association between rs978739 and the longevity phenotype, but no evidence of association between rs978739 and the longevity phenotype was observed according to the additive or dominant model [11].

The use of meta-analyses has become an important part of genetic research, mainly to reconcile previously conducted studies that have given inconsistent results [12]. Therefore, in the present paper a meta-analytic approach to quantitatively synthesize the possible effect of the previous variants and possibly, to reconcile the inconsistencies has been used. In the present study TAS2R38 variants were also analysed in the Cilento population for their possible association with longevity and the obtained data have been included in the relative meta-analysis.

Furthermore, as it is believed that Apolipoprotein E(ApoE) alleles, which strongly influence longevity in most populations [5, 6], might interact with other genes to influence successful aging and longevity [13], we have analysed the association of TAS2R38 with longevity in conjunction with the Translocase of outer mitochondrial membrane 40 homolog (TOMM40), in linkage disequilibrium with APOE [14]. The TOMM40 rs2075650 SNP was analysed, since it showed evidence of association with the longevity phenotype [14], and it is correlated with rs429358, one of the SNPs defining the APOE-ε4 allele together with rs7412 (rs2075650:rs429358 *r*^2^ = 0.56/*D*' = 0.81, rs2075650:rs7412 *r*^2^ = 0.01/*D*' = 0.84) in non-Finnish European populations [15], both absent among the SNPs on the used chip [16].

## Materials and methods

### Study participant

The analysis has been performed on data previously obtained with genome-wide association study (GWAS) on a population of LLIs (age range 90–109 years) and young controls (age range 18–45 years). They had been recruited as part of the Southern Italian Centenarian Study (SICS) [16]. The LLIs were thoroughly investigated for demographic characteristics, medical history (past and present diseases), level of independence and cognitive status. All subjects donated blood samples for DNA study and gave written informed consent to the study, which was approved by Ethical Committee of Multimedica Hospital. All methods were performed in accordance with the relevant guidelines and regulations. The study was conducted in accordance with the ethical principles that have their origin in the Declaration of Helsinki.

### Genotyping

Genotyping had been carried out with the Infinium II Assay-HumanHap BeadChip 317 K duo system using standard protocols of the Illumina HumanHap 317 Duo workflow (Illumina, San Diego, CA). All genotypes had been evaluated using a quantitative quality score called GenCall score. The initial screening dataset was represented by 466 LLIs and 624 controls. After quality control, it was looked for evidence of genetic population stratification on a subset of 454 LLIs and 591 young controls. After outliers were removed (*n* = 82), the final GWAS dataset was composed of 963 samples, of which 410 were cases (age range 90–109 year male/female ratio, 1.4, and 553 were controls (age range 18–48 year male/female ratio 1.56).

Genotypes pre-phasing was performed by the Shapeit2 software increasing the number of states up to 500 to improve the phasing accuracy and using the reference genetic map for chromosome 7 [17]. The deriving haplotypes were then used as input during the imputation of unobserved genotypes that was performed by the Impute2 software [18]. Haplotypes generated by the Impute2 software during the imputation process by the phase option were then used for the analysis of the 3-SNPS haplotypes mapping to the TAS2R38 gene. Statistical power calculations were performed by the Quanto software (https://biostats.usc.edu/Quanto.html) based on results described in Melis et al. [9] except for the sample size of the replication cohort [16]. The significance threshold was set to *α* = 0.05. The TOMM40 rs2075650 was genotyped as previously described [14, 16].

### Statistics

The possible association of haplotypes and genotypes with longevity was analysed by the Pearson Chi-square test for independence (3 × 2 contingency tables, 2 degrees of freedom and 4 × 2 contingency tables, 3 degrees of freedom). The Fisher’s exact test, univariate and multivariate logistic regression were also used to test for association between haplotypes, the TOMM40 rs2075650 SNP and the longevity phenotype in the SICS cohort, using functions implemented in the R statistical software tool (www.r-project.org). To this aim, rs2075650 and haplotypes were coded according to the number of G alleles (rs2075650) and PAV, AVI haplotypes (TAS2R38).

### Study design of meta-analysis

The primary source of the studies addressing the role of the bitter taste receptor polymorphisms in longevity was the PUBMED database (from inception to August 2020) limited to English language literature. The medical subject headings used were “bitter taste receptor” [OR] TAS2R [OR] “bitter receptor” [OR] TAS2R [AND] “polymorphisms” [AND] “longevity”. The retrieved abstracts were read to identify studies examining the genotype association between polymorphisms within the TAS2R genes with longevity. A manual search of references cited in published articles was also performed. The studies were read in their entirety to assess their appropriateness for inclusion in the meta-analysis. Criteria for the inclusion in the analysis were: case–control studies, available genotype or allele, and control population in Hardy–Weinberg equilibrium. Three studies were finally retrieved and included in the meta-analysis [9–11]. Data on the role of TAS2R16 gene (one polymorphism, rs978739) and the TAS2R38 gene (3 SNPs defining the genotype named PAV, that is the functional form, or the genotype AVI, the non-functional variant) were available for meta-analysis. Extraction of the data was independently performed by two readers (DDB and CC) who compared results and agreed on a consensus disagreements were resolved by discussion. Concerning the TAS2R38 gene, the data depicted in Tables 2 and 3 of the present study were also included.

### Statistics of meta-analysis

To analyse data, Review Manager, version 5.2, a statistical software package for managing and analysing all aspects of a Cochrane Collaboration systematic review was used. The overall Odds Ratio (OR) of genotypes in both control group and LLIs (age cut-off, 85 years in the Campa study/90 years in the Malovini and the Melis studies) was estimated using a model based on random effects assumptions, which is more conservative one than the fixed effects model. The random effects model uses weights that incorporate both the within-study and between-study variance [19]. The 95% confidence interval (CI) of the OR was also calculated.

For the TAS2R38 gene, two aggregated genotype data (AVI/AVI + PAV/AVI) were compared to the functional genotype variant in homozygosity (PAV/PAV). A comparisons between the haplotypes (AVI vs. PAV) as well as between the two homozygous genotypes (AVI/AVI vs. PAV/PAV) was also made.

For the TAS2R16 gene, two aggregated genotype data (T/C + C/C) were compared to the genotype putatively associated with longevity in homozygosity (TT). A comparisons between the alleles (C vs. T) was also performed as well as between the two homozygous genotypes (C/C vs. T/T).

Table 1 shows these different populations, by location, with age distribution by size and genotypes.

**Table 1:** Demographic and genetic features of the cases and control cohorts

Population	Cases (N)	Age range	PAV/PAV (N)	T/T (N)	Controls (N)	Age range	PAV/PAV (N)	T/T (N)
Cilento	410	90–109	98	186	553	18–45	137	252
Calabria	348	85–106	331^a^	185	593	20–84	585^a^	245
Sardinia	94	90–105	32	N.D	181	18–35	41	N.D
Sardinia					98	36–85	18	N.D

Location, age and number of subjects positive for the genotypes reported to be associated with longevity

^a^For Calabria’s LLIs and Controls were available only haplotype data

## Results

### Genotyping

The TAS2R38 haplotypes were defined by the following three SNPs: rs10246939, rs1726866, and rs713598. Of these SNPs, rs10246939 and rs1726866 were represented on the Illumina genotyping chip, while rs713598 was imputed (info score = 0.957). The distributions of haplotypes and genotypes in LLIs and controls are reported in Tables 2 and 3, respectively. By Chi-square test, no significant differences between LLI and control frequencies were observed.

**Table 2:** Haplotype frequencies of polymorphisms of TAS2R38 gene in LLIs and younger controls from Cilento (South Italy)

Haplotypes	LLIs		Controls	
	N	%	N	%
PAV	398	48.54	555	50.18
AVI	392	47.80	515	46.57
RARE	30	3.66	36	3.25

The Chi-square statistic (3 × 2) is 0.635. The *p* value is 0.727969. The result is not significant at *p* < 0.05

**Table 3:** Genotype frequencies of polymorphisms of TAS2R38 gene in LLIs and younger controls from Cilento (South Italy)

Genotypes	LLIs		Controls	
	N	%	N	%
PAV/PAV	98	23.90	137	24.77
PAV/AVI	186	45.37	263	47.56
AVI/AVI	98	23.90	118	21.34
RARE	28	6.83	35	6.33

The Chi-square statistic (4 × 2) is 1.0964. The *p* value is 0.777952. The result is not significant at *p* < 0.05

Furthermore, as stated in the Introduction, we tested whether the TOMM40 SNP rs2075650 (Table 4) could influence the association between the PAV/AVI haplotypes and the longevity phenotype. Rs2075650 showed evidence of association with the longevity phenotype in the Cilento cohort according to the genotypic model (global *p* = 0.0272). In particular, carriers of the heterozygote AG genotype had a statistically significant reduction in terms of probability of being LLIs compared to AA genotypes (OR AG vs. AA = 0.56, 95% CI = 0.35–0.88, *p* = 0.0144), while no statistically significant difference has been observed between GG and AA genotypes (OR GG vs. AA = 1.71, 95% CI = 0.37–8.71, *p* = 0.485). When rs2075650 was included in a multivariate logistic regression model with the PAV/AVI haplotypes, no evidence of association between haplotypes and the phenotype was observed neither according to an additive model nor by comparing genotypes configurations (*p* > 0.05, Table 4).

**Table 4:** Multivariate logistic regression including rs2075650 SNP and TAS2R38 haplotypes

Variable	OR	L95	U95	*p*
Haplotype: genotypic model				
rs2075650 = AG	0.56	0.34	0.88	0.013
rs2075650 = GG	1.80	0.39	9.21	0.444
Haplotype = AVI/AVI	1.08	0.61	1.91	0.792
Haplotype = PAV/AVI	0.89	0.52	1.53	0.669
Haplotype = PAV/PAV	0.91	0.52	1.61	0.751
Haplotype: additive model				
rs2075650 = AG	0.56	0.35	0.88	0.015
rs2075650 = GG	1.73	0.38	8.85	0.473
Haplotype = PAV	0.88	0.54	1.44	0.595
Haplotype = AVI	0.94	0.58	1.54	0.796

Variable: variable included in the multivariate model (genotypic model: Haplotype  = AVI/AVI: subjects carrying the AVI haplotype on both chromosomes, Haplotype = PAV/AVI: subjects carrying PAV on one chromosome and AVI on the other chromosome, Haplotype = PAV/PAV: subjects carrying the PAV haplotype on both chromosomes, carriers of the rare haplotype have been used as baseline value; Additive model: Haplotype = PAV: number of PAV haplotypes by subject, Haplotype = AVI: number of AVI haplotypes by subject); *OR* odds ratio for longevity, *L95* lower bound of the OR 95% confidence interval; U95% upper bound of the OR 95% confidence interval

### Meta-analysis

Two studies on the association between the TAS2R38 PAV haplotype and longevity were identified by search strategy. The first one demonstrated that this haplotype did not show a statistically significant association with longevity (583 controls 20–85 years vs. 340 LLIs 86–106 years). Due to the lack of significance, the authors only reported haplotype data [10]. Subjects had been recruited between 1994 and 2008 in Calabria. The authors chose 85 years as a cut-off point, because, in their opinion, genetic factors contribute to the variation in human life span minimally before age 60 years and most profoundly from age 85 years onwards. Study participants, their parents, and grandparents were all born in Calabria, as ascertained from population registers [10].

Instead, a significant association of the PAV/PAV genotype with longevity was found in the second paper [9]. Three hundred seventy-three subjects were included in the study. They were divided in three groups based on their age and area of Sardinia island (Italy), where they were recruited. The Longevity Blue Zone cohort (*n* = 94) (age ranging from 90 to 105 years) included subjects recruited in the central‒eastern area (Ogliastra/Barbagia) of Sardinia; the Cagliari young cohort (*n* = 181) (age ranging from 18 to 35 years) included subjects recruited in the area of the city of Cagliari (Sardinia); the Cagliari cohort including middle-aged adults and older adults (*n* = 98) (age ranging from 36 to 85 years) with subjects recruited in the same area of young cohort [9]. This zone of Sardinia is one of the five world Blue Zone, i.e., zones of exceptional longevity. Blue Zone populations are geographically and/or historically isolated and live in an ideal environment for the emergence of long-lived phenotypes at the population level. People living in these areas present different cultural traditions but a common characteristic, the healthy lifestyle. Some of them are vegetarians, others follow occasionally fasting and live the life with a positive mood, socially engaged and physically active [20].

Finally, in the present study, it was not found any significant association of PAV with longevity (553 controls, 18–45 years vs. 410 LLIs, 90–109 years) (Tables 2, 3).

**Fig. 1:**
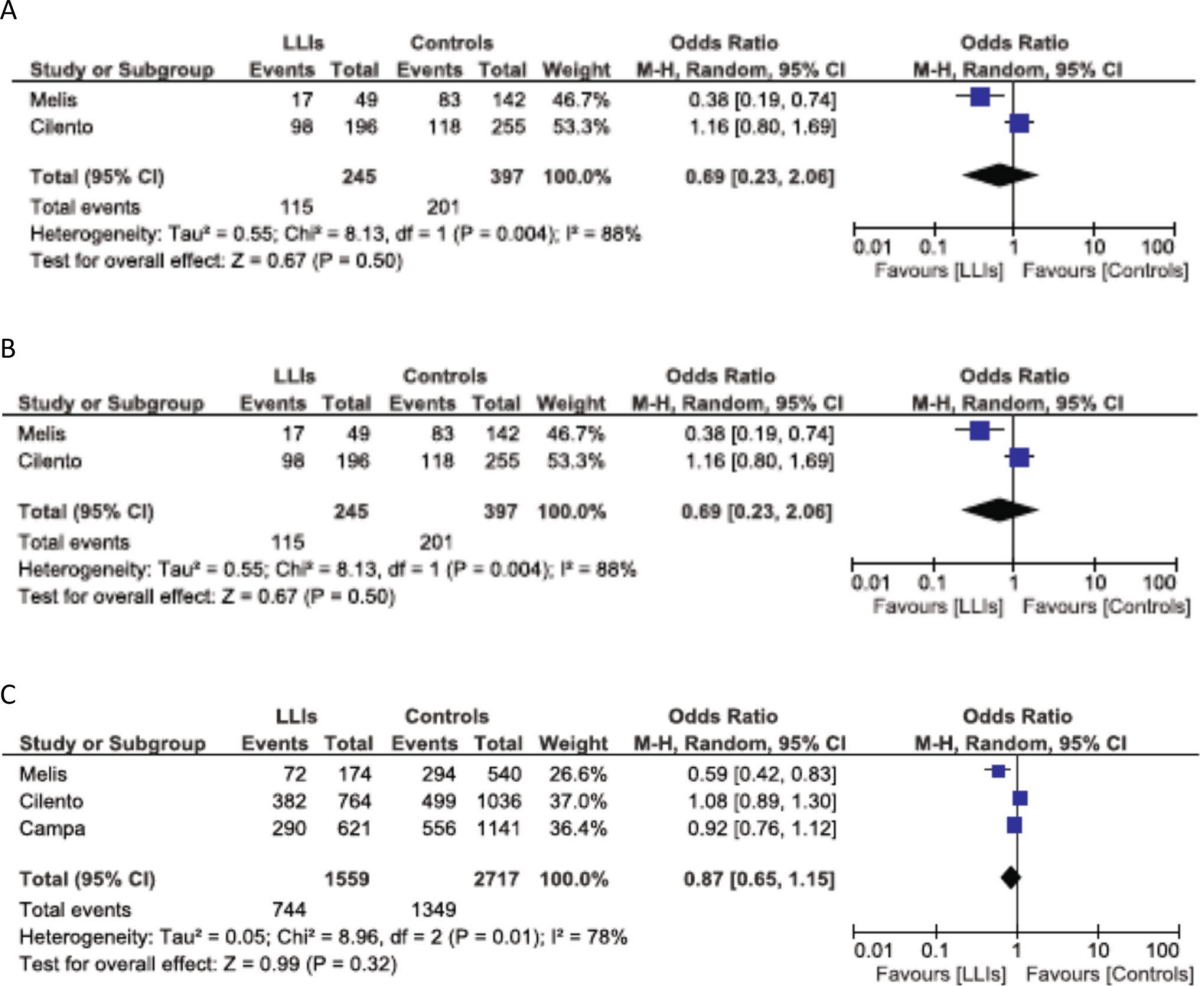
Meta-analysis of the two case–control studies (three for C) of the TAS2R38 polymorphism and longevity using the random effects model. The OR and 95% CI for the effect of the various genotypes/haplotypes on longevity are plotted on the graphs. Cilento refers to data presented in Table 1a, b. **a** AVI/AVI + PAV/AVI vs. PAV/PAV, **b** AVI/AVI vs. PAV/PAV, **c** AVI vs. PAV

Figure 1a–c depicts the meta-analysis of PAV/AVI haplotypes and genotypes (rare haplotypes and genotypes were not taken into account). The pooled summary OR for the genotypic comparison between the AVI/AVI + PAV/AVI vs. PAV/PAV is 0.73 (95% CI 0.34–1.55) not reaching statistical significance using the random-effects model (Fig. 1a). The pooled summary OR for the genotypic comparison between the AVI/AVI vs. PAV/PAV is 0.69 (95% CI = 0.23–2.06), not reaching statistical significance using the random-effects model (Fig. 1b). The pooled summary OR for the allelic comparison between the AVI vs. PAV is 0.87 (95% CI = 0.65–1.15), not reaching statistical significance according to the random-effects model (Fig. 1c).

Two studies on the association between the TAS2R16 rs978739 and longevity were identified by search strategy. The first one demonstrated that one polymorphism, rs978739, situated 212 bp upstream of the TAS2R16 gene, shows a statistically significant association with longevity (583 controls 20–85 years vs. 340 LLIs 86–106 years) [8]. In the second paper it was not, instead, found any statistically significant association of this SNP with longevity (553 controls 18–45 years vs. 410 LLIs 90–109 years) [9].

**Fig. 2:**
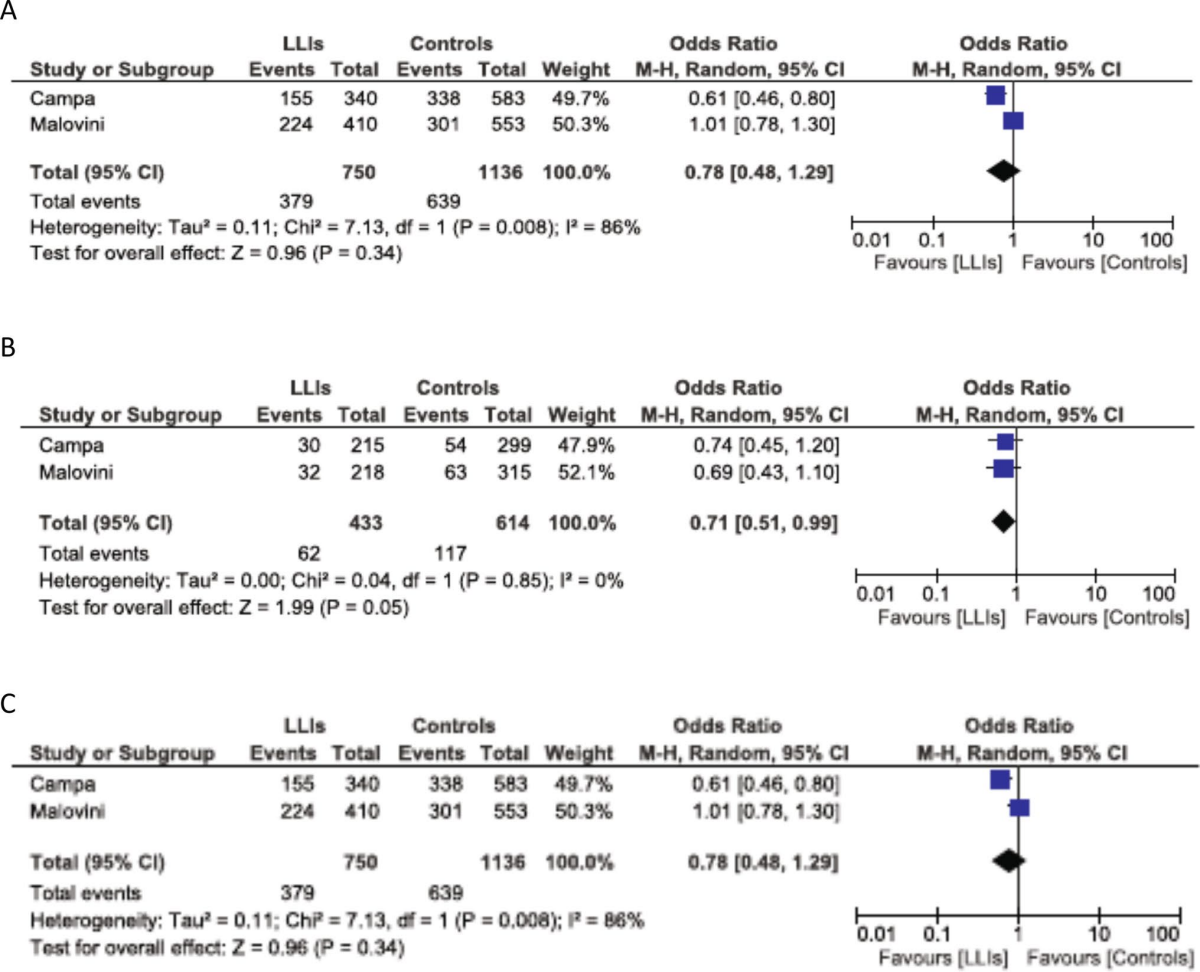
Meta-analysis of the two case–control studies of the TAS2R16 polymorphism and longevity using the random effects model. The OR and 95% CI for the effect of the various genotypes/alleles on longevity are plotted on the graphs. **a** CC + CT vs. TT; **b** CC vs. TT; **c** C vs. T

Figure 2a–c depicts the meta-analysis of SNP alleles coded according to the forward strand (hg19). The pooled summary OR for the genotypic comparison between the CT + CC vs. TT is 0.78 (95% CI = 0.48–1.29) without statistical significance using the random-effects model (Fig. 2a). The pooled summary OR for the genotypic comparison between the CC vs. TT is 0.71 (95% CI = 0.51–0.99) reaching borderline statistical significance (*p* = 0.05) using the random-effects model (Fig. 2b). The pooled summary OR for the allelic comparison between the C vs. T is 0.78 (95% CI = 0.48–1.29) without statistical significance using the random-effects model (Fig. 2c).

## Discussion

LLIs, i.e., people belonging to the 5 percentile of the survival curve, are genetically predisposed to reach extreme ages, as suggested by family clusters of extreme longevity. There are many possible candidate genes for human longevity, however, of the many genes tested, only APOE and FOXO3 survived to association in replication studies. In fact, it is necessary to validate in other studies genes suggested to be associated with longevity [5, 21]. Thus, the aim of the present paper was to validate the reported association of taste receptor with longevity.

The TAS2R38 protein has two common variants that differ in the amino acid residues at positions 49, 262, and 296 [9]. No association was found between TAS2R38 polymorphisms and longevity in Cilento population, as well as performing the meta-analysis with data of the other studies.

Concerning TAS2R16 gene, it has been studied the polymorphism of the position 212 bp upstream (rs978739), most likely located in the promoter region for TAS2R16, thus suggesting a critical regulation role [10]. T/T, significantly associated with longevity in the Calabria population, maintained its significance in the meta-analysis with data from Cilento population, that were not significant in the previously published study. Therefore, present results strengthen the suggestion that TAS2R16 genotype T/T is associated with longevity in South Italy.

TAS2R belong to the superfamily of seven-transmembrane G protein–coupled receptors. TAS2R are located in taste buds of the tongue, where they initiate bitter taste perception. However, it has becoming clear that taste receptors are widely expressed throughout the body and mediate diverse non-tasting functions. Cumulative evidence indicates that TAS2R mediate a variety of functions in non-lingual tissues and may underlie several human diseases or disorders. It has also become apparent that taste receptor polymorphisms are associated with human traits and disorders [1–3].

In particular, TAS2R-mediated signalling contributes to the innate immune-inflammatory responses in the epithelia of the organs connected to the external environment. Most of the studies related to their role in immune-inflammatory responses have been focused on the respiratory system. In fact, bitter taste receptors might be considered as sentinels of defence against infection in the airway, where they play a role as detectors of pathogens, since they should be able to respond to bitter molecules released by pathogens in the mucosal environment. It is reasonable to speculate that mechanisms similar are used to monitor microbiota in the gut and that functional polymorphisms influence their activity [1–3]. In addition, it has been shown that the bitter taste receptors are expressed in neutrophils and monocytes, as well as in resting and activated human lymphocytes, and have been claimed to mediate an anti-inflammatory effect [22, 23]. Because of the well-known role of both an efficient immune response and an optimal control of inflammation in the attainment of longevity [24, 25], these mechanisms might explain the association of TAS2R with longevity.

On the other hand, TAS2R genes seem to have been under balancing selection for a long time before the emergence of modern humans in Africa. Their role was most likely to prevent consumption of dangerous raw foods, taking advantage of a wide spectrum of sensations of bitterness [4]. Therefore, diet-related mechanisms can be responsible for association with longevity. As an example that concerns TAS2R38, the frequency of vegetable and fat intake, is reciprocally regulated by the functional haplotype PAV, i.e., an increased intake of vegetables and decreased intake of fat [26], so favouring the intake of anti-inflammatory foods [27], known to confer protection against inflammatory age-related diseases [25], including cardiovascular disease ones, the most important death cause in the Western World [3].

These findings strengthen previous suggestions of an association between genetic variants of TAS2R16 gene and human longevity, highlighting the role of the G protein-coupled receptor in the molecular physiological mechanisms involved in the biological process of ageing and longevity.

## References

[1] Lu P, Zhang CH, Lifshitz LM (2017). Extraoral bitter taste receptors in health and disease. J Gen Physiol.

[2] Roper SD, Chaudhari N (2017). Taste buds: cells, signals and synapses. Nat Rev Neurosci.

[3] Simpson KL, Haines DE, Mihailoff GA (2018). Olfaction and taste. Fundamental neuroscience for basic and clinical applications.

[4] Valente C, Alvarez L, Marques PI (2018). Genes from the TAS1R and tas2r families of taste receptors: looking for signatures of their adaptive role in human evolution. Genome Biol Evol.

[5] Caruso C, Aiello A, Accardi G (2019). Genetic signatures of centenarians: implications for achieving successful aging. Curr Pharm Des.

[6] Accardi G, Aprile S, Candore G (2019). Genotypic and phenotypic aspects of longevity: results from a Sicilian survey and implication for the prevention and treatment of age-related diseases. Curr Pharm Des.

[7] Tepper BJ (2008). Nutritional implications of genetic taste variation: the role of PROP sensitivity and other taste phenotypes. Annu Rev Nutr.

[8] Lee RJ, Cohen NA (2015). Role of the bitter taste receptor T2R38 in upper respiratory infection and chronic rhinosinusitis. Curr Opin Allergy Clin Immunol.

[9] Melis M, Errigo A, Crnjar R (2019). TAS2R38 bitter taste receptor and attainment of exceptional longevity. Sci Rep.

[10] Campa D, De Rango F, Carrai M (2012). Bitter taste receptor polymorphisms and human aging. PLoS ONE.

[11] Malovini A, Accardi G, Aiello A (2019). Taste receptors, innate immunity and longevity: the case of TAS2R16 gene. Immun Ageing.

[12] Di Bona D, Accardi G, Virruso C (2014). Association of Klotho polymorphisms with healthy aging: a systematic review and meta-analysis. Rejuven Res.

[13] Raichlen DA, Alexander GE (2014). Exercise, APOE genotype, and the evolution of the human lifespan. Trends Neurosci.

[14] Sebastiani P, Solovieff N, Dewan AT (2012). Genetic signatures of exceptional longevity in humans. PLoS ONE.

[15] https://analysistools.cancer.gov/LDlink/?tab=ldpair, accessed on 28 Sep 2020

[16] Malovini A, Illario M, Iaccarino G (2011). Association study on long-living individuals from Southern Italy identifies rs10491334 in the CAMKIV gene that regulates survival proteins. Rejuven Res.

[17] O'Connell J, Gurdasani D, Delaneau O (2014). A general approach for haplotype phasing across the full spectrum of relatedness. PLoS Genet.

[18] Howie BN, Donnelly P, Marchini J (2009). A flexible and accurate genotype imputation method for the next generation of genome-wide association studies. PLoS Genet.

[19] DerSimonian R, Laird N (1986). Meta-analysis in clinical trials. Control Clin Trials.

[20] Poulain M, Caruso C (2019). Individual longevity versus population longevity. Centenarians. An example of positive biology.

[21] Puca AA, Spinelli C, Accardi G (2018). Centenarians as a model to discover genetic and epigenetic signatures of healthy ageing. Mech Ageing Dev.

[22] Tran HTT, Herz C, Ruf P (2018). Human T2R38 bitter taste receptor expression in resting and activated lymphocytes. Front Immunol.

[23] Orsmark-Pietras C, James A, Konradsen JR (2013). Transcriptome analysis reveals upregulation of bitter taste receptors in severe asthmatics. Eur Respir J.

[24] Puca AA, Ferrario A, Maciag A (2018). Association of immunoglobulin GM allotypes with longevity in long-living individuals from Southern Italy. Immun Ageing.

[25] Aiello A, Farzaneh F, Candore G (2019). Immunosenescence and its hallmarks: How to oppose aging strategically? A review of potential options for therapeutic intervention. Front Immunol.

[26] Calancie L, Keyserling TC, Taillie LS (2018). TAS2R38 predisposition to bitter taste associated with differential changes in vegetable intake in response to a community-based dietary intervention. G3 (Bethesda).

[27] Accardi G, Shivappa N, Di Maso M (2019). Dietary inflammatory index and cancer risk in the elderly: a pooled-analysis of Italian case-control studies. Nutrition.

